# Difficult removal of an endobronchial foreign body: pen cap aspiration: a case report

**DOI:** 10.1186/s13256-025-05334-8

**Published:** 2025-06-19

**Authors:** Hujun Wu, Xiaofen Tao, Liqin Ke, Lei Wu

**Affiliations:** 1 Department of Pediatric Pulmonology, The Children’s Hospital, National Clinical Research Center for Child Health, Zhejiang University School of Medicine, 3333 Binsheng Road, Hangzhou, 310052 Zhejiang China; 2 Department of Endoscopy Center, The Children’s Hospital, National Clinical Research Center for Child Health, Zhejiang University School of Medicine, Hangzhou, 310052 Zhejiang China

**Keywords:** Foreign body aspiration, Pen cap, Bronchoscopy

## Abstract

**Background:**

Foreign body aspiration represents an important cause of morbidity and mortality during childhood. Foreign body aspiration is often missed clinically, especially in some children whose symptoms and signs are not obvious. A neglected aspirated foreign body can last for years, leading to recurrent pneumonia in mild cases and the potential to be life-threatening in severe cases. We herein report a case of difficult foreign body removal in the left upper lobe to help us better understand the risk of foreign body aspiration.

**Case presentation:**

The patient was a 12-year-old boy of East Asian ethnicity who complained of a history of magnetic bead aspiration 17 days earlier. He was admitted to hospital due to fever 5 days previously with a mild cough and chest pain. Chest computed tomography confirmed a bronchial foreign body. Due to the special location of the foreign body impaction and prolonged retention, it was very difficult to remove. We successfully removed the peripheral granulation tissue with the help of a combination of various bronchoscopic interventional techniques and finally removed the foreign body. However, to our surprise, the foreign body was not a magnetic bead but a pen cap.

**Conclusion:**

Foreign body aspiration is common in childhood and requires early recognition and treatment to avoid complications that can be very serious or even fatal. Bronchoscopy is the gold standard for the diagnosis of foreign body aspiration. For difficult foreign body removal, bronchoscopic intervention technology can be useful.

## Background

Foreign body aspiration is most common in young children, especially in children aged 1–3 years, with a peak incidence between 1 and 2 years of age [1]. Previous research has shown that tracheobronchial foreign body accounts for 7.9–18.1% of accidental injuries in children aged 0–14 years in China [2]. There are no official statistics on mortality due to bronchial foreign bodies. According to previous literature, the in-hospital mortality rate for respiratory foreign bodies under 5 years of age ranges from 0.36% to 2.75% [3–5].

Clinical features depend on the type, location, and size of the foreign body, as well as the duration and degree of obstruction. Common symptoms of foreign body aspiration include coughing, choking, wheezing, stridor, and dyspnea. The most common sign is diminished or absent breath sounds [6–8]. However, a considerable number of patients found no abnormalities in physical examination. Mallick reported that if the diagnosis of foreign body inhalation is delayed (> 24 h), the incidence of complications such as dyspnea, asphyxia and death, pneumothorax, lung infection, atatasis, and obstructive emphysema is greatly increased [9]. In severe cases, thoracotomy may be required, thus timely diagnosis is critical.

In our research, we reviewed a foreign body in the upper left lung, which helped us better understand the risk, diagnosis, and treatment of foreign body aspiration.

## Case presentation

**Fig. 1:**
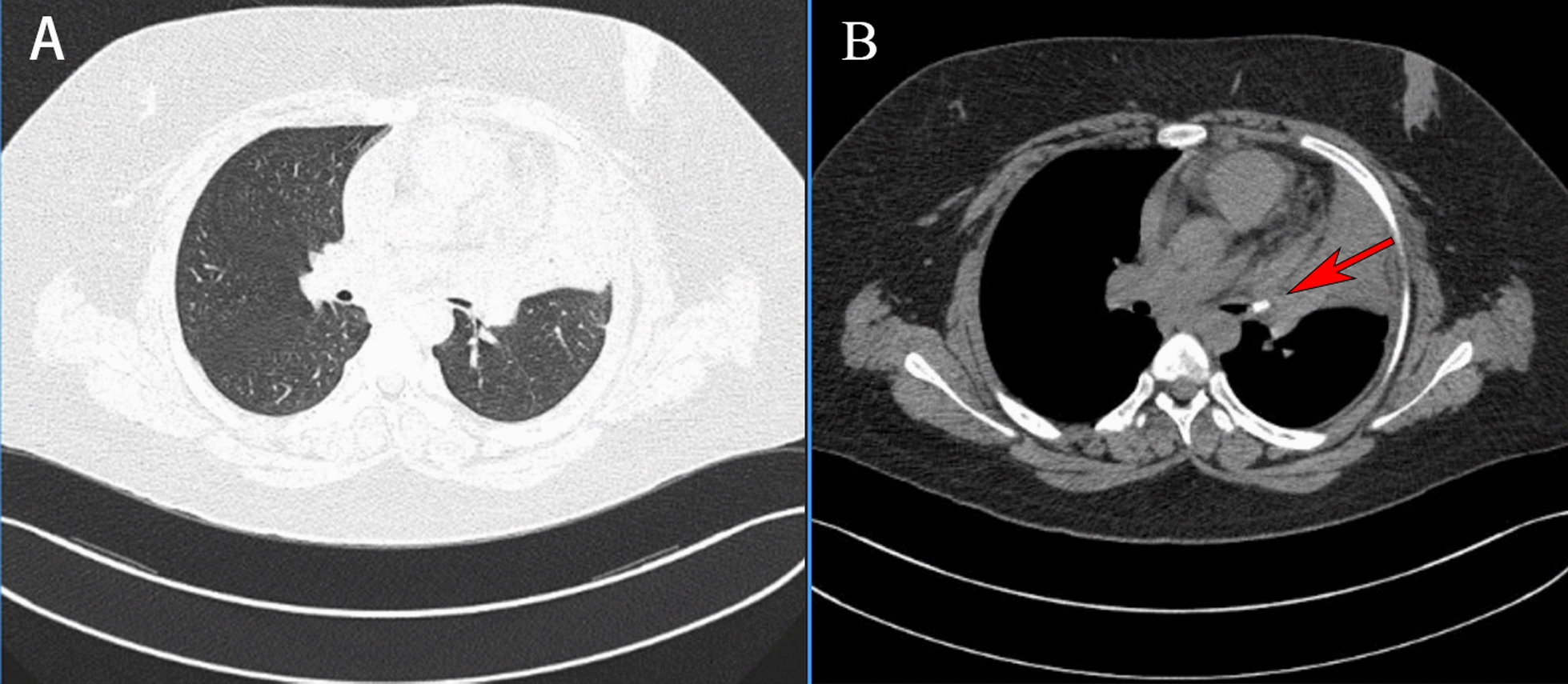
Chest computed tomography images of the child. **A**, **B** chest computed tomography showing a high-density shadow (red arrow) in the main bronchial lumen of the left superior lobe with partial atelectasis

The patient was a 12-year-old boy of East Asian ethnicity who complained of a history of magnetic bead aspiration 17 days earlier. Initially, due to the negligence of the parents, the child did not come to the hospital in the early stage, and 5 days ago, began to have fever with cough and chest pain. He was admitted to the local hospital and chest computed tomography (CT) showed a high-density shadow in the main bronchial lumen of the left superior lobe with partial atelectasis, and a foreign body was considered (Fig. 1). Because of the limited medical care in the area, the child was transferred to our hospital.

**Fig. 2:**
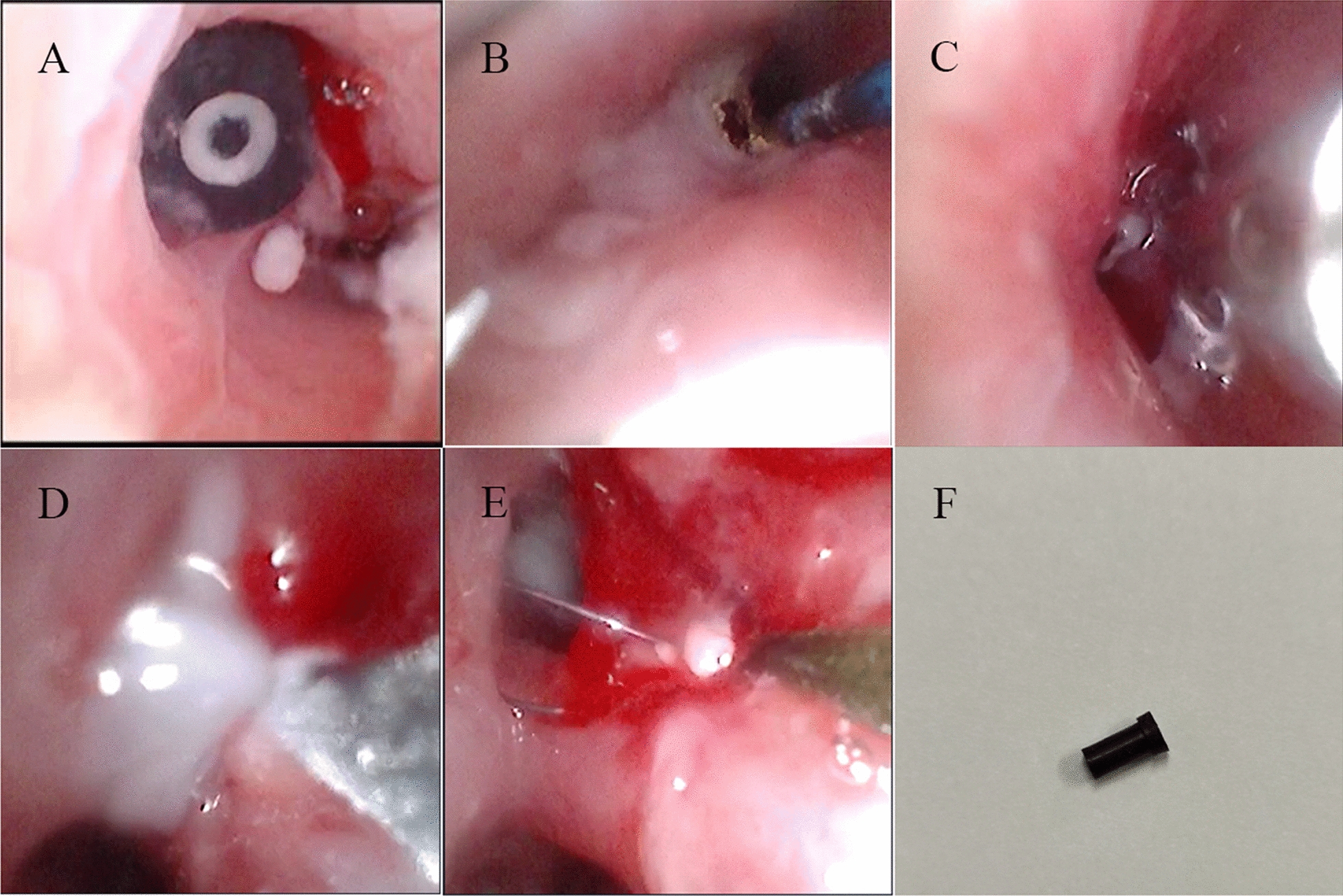
Endoscopic intervention assisting removal of the foreign body. **A**–**F** Black foreign body can be seen in the opening of the left lingual lobe. **B**–**D** Granulation tissue and large amount of white purulent secretions at the left upper leaf opening. The granulation tissue and phlegm were treated with holmium laser, biopsy forceps, and cryosurgery. **E** Foreign body basket to remove foreign body

The day after the patient’s admission, we performed the first bronchoscopy, and saw a large granulation forming in the upper left lung opening. Surgery was made trickier by the fact that the foreign body was long embedded and in the relatively difficult left upper lobe of the lung. We cleaned the granulation tissue by biopsy forceps and holmium laser, but the granulation root was too large to be completely removed. We then scheduled a second surgery 2 days later, during which we administered intravenous steroids to reduce granulation tissue swelling and ceftriaxone for anti-infective treatment. During the second operation, we found that there was a large amount of viscous discharge from the left main bronchus, which was difficult to be cleared by negative pressure suction. To solve this problem, we used cryotherapy. By means of repeated freezing and removal, we found large granulation formation and surface necrosis in the left upper lobe. Biopsy forceps were thus used to clean the granulation tissue. After cleaning, we found that the opening of the left upper lobe was narrow, and a black foreign body was visible in the opening of the lingual lobe. However, it was still difficult to remove the foreign body after several attempts of forceps. Finally, we performed balloon dilation for the child, and after enlarging the bronchial lumen, we successfully used the foreign body basket to collect foreign body. Interestingly, the foreign body removed was not a magnetic bead but a pen cap (Fig. 2).

## Discussion

Foreign body aspiration is one of the common emergencies in emergency room, which often endangers the life of children. The common foreign bodies can be classified in two types: edible and inedible foreign bodies. Edible foreign bodies are common in young children, especially in children between 1 and 3 years of age (mean age of 1.8 years), whereas inedible foreign bodies are common in older children (mean age of 5.22 years) [10]. Common inedible foreign bodies include whistles, pen caps, stones, screws, magnetic beads, bones, plastic parts of toys, etc. Jiaqiang *et al*. conducted a survey that indicated that 2.65% of all children had a history of pen cap aspiration [11], and an Indian study showed that pen caps are the second most important factor in the inedible group [12]. Similar to most studies, our result showed that a pen cap was inhaled into the bronchus and lodged in the lingual segment of the left superior lung. Interestingly, the chief complaint of the parents was aspiration of magnetic beads. Although the objects were different, it ultimately led to the same outcome. The warning from this case is that the history of foreign body inhalation had crucial significance for clinical diagnosis.

In our study, we reported a case of foreign body in the lingual segment of the left superior lung. Due to the foreign body being embedded in a special place, its retrieval was performed with resultant great difficulty. At present, there are few reports regarding the use of flexible bronchoscope to remove foreign bodies in this area. However, trachea foreign body in distal left upper lung were not uncommon. In general, distal bronchopulmonary foreign bodies were often difficult to reach using rigid bronchoscope [13]. Although the flexible bronchoscope could reach the peripheral bronchioles, in our case, the visual field was greatly affected by the formation of granulation tissue and lumen stenosis due to the prolonged stay of foreign bodies in the airway. This made it harder for us to removal of the foreign object. In addition, the insertion of biopsy forceps limited the flexibility of the flexible bronchoscope during operation. All of these conditions could lead to the failure of the procedure. Ding and Ünal *et al*. described that when foreign bodies remain in the airway for more than 7 days, mucosal edema and strong inflammatory response are likely to occur, and surgery-related morbidity will be increased, ultimately increasing difficulty of treatment [14, 15]. Fortunately, we were eventually able to remove the foreign body. Therefore, we recommend early and active bronchoscopy for children with a history of foreign body aspiration. This may effectively reduce the occurrence of complications.

## Conclusion

With the continuous development of bronchoscopy technology, the status of flexible bronchoscopy in the removal of pulmonary foreign bodies is also improving. With the help of various technologies, the failure rate of children’s surgery is also decreasing. However, the high mortality and complications associated with foreign body inhalation are also related to the size, blocked site, and duration of foreign body aspiration, so the history of foreign body inhalation is more important in diagnosis. Early diagnosis will greatly reduce the death and complications of children in the later stage.

## Data Availability

The datasets used and/or analyzed during the current study are available from the corresponding author upon reasonable request.
